# Predictors of adult ICU mortality: a retrospective study at two government hospitals in Ethiopia

**DOI:** 10.1038/s41598-026-43206-3

**Published:** 2026-03-07

**Authors:** Shimels Getaneh Weldemedhn, Behaylu Tesfamaryam Hagos, Alyas Muche Kebede, Wegderes Bogale Gebresilassie, Melaku Tsediew Berhanu, Demmelash Gezahegn Nigatu

**Affiliations:** 1 Addis Ababa University, College of Health Science, Department of Emergency and Critical Care Medicine, Addis Ababa, Ethiopia; 2 Department of Internal Medicine, Debre Birhan University, Asrat Woldeyes Health Science Campus, Debre Berhan, Ethiopia; 3 Department of Pediatric and Child Health, Debre Birhan University, Asrat Woldeyes Health Science Campus, Debre Berhan, Ethiopia; 4 Department of Emergency and Critical Care Medicine, Debre Birhan University, Asrat Woldeyes Health Science Campus, Debre Berhan, Ethiopia

**Keywords:** Predictors, ICU mortality, Charlson comorbidity index, Hospital-acquired infection, Ethiopia, Diseases, Health care, Medical research, Risk factors

## Abstract

The number of life-threatening conditions requiring admission to intensive care units has increased substantially in low-income countries, partly due to the expansion of hospital services. In Ethiopia, ICU mortality rates vary across regions. However, evidence regarding the magnitude of ICU mortality and its associated predictors remains limited and inconclusive. To assess the magnitude of the mortality rate and its predictors among hospitalized adult patients A two-center retrospective cross-sectional study was conducted among patients admitted to the ICU between December 1, 2023, and May 30, 2024. Data were collected using a pretested, structured questionnaire. The completed data were gathered via a web link developed using Kobo Toolbox (kobtoolbox.org), then coded, manually verified for completeness, and exported to SPSS version 27 for analysis. Descriptive statistics and logistic regression analyses were performed to evaluate the data. A total of 309 patient charts were reviewed. The median ICU stay was 5 days. The leading causes of ICU admission were postoperative conditions, septic shock, stroke, and congestive heart failure. The most common causes of death were septic shock, stroke, head trauma, and acute respiratory distress syndrome (ARDS). The overall mortality rate among ICU-admitted patients was 46.3%. A higher Charlson Comorbidity Index score, the need for mechanical ventilation at admission, and the presence of hospital-acquired infections were significantly associated with ICU mortality. Compared with some developed countries, the observed mortality rate in this cohort was higher. The findings of the present study indicate that hospital-acquired infections, the Charlson Comorbidity Index, and the need for mechanical ventilation were all significantly associated with mortality among intensive care unit patients.

**Supplementary Information:**

The online version contains supplementary material available at 10.1038/s41598-026-43206-3.

## Introduction

 The intensive care unit (ICU) is the hospital department dedicated to the care of critically ill patients. It relies on a specialized, multidisciplinary team and advanced medical technology to provide optimal care for patients who are typically unstable and at high risk of mortality. These patients require continuous monitoring of a wide range of clinical and laboratory parameters, which directly influence their clinical course and guide the healthcare team’s decision-making^1–3^.

Worldwide, the percentage of patients admitted to intensive care units (ICUs) ranges from 1% to 40% or more^4^. The reasons for ICU admission vary globally; however, most available data indicate that the need for and utilization of ICU increased^5^.

Studies have shown that ICU patient outcomes were influenced by a variety of factors, including disease type and severity, patient age, presence of comorbidities or multi-organ failure, the need for mechanical ventilation, length of ICU stay, ICU-acquired complications, and the spread of antimicrobial-resistant microorganisms^6–8^.

In critical care, several scoring systems are available to assess severity and predict outcomes^9^. Outcome prediction models are typically categorized as disease-specific or generic^10,11^. These methods usually assign points based on the severity of illness and claim to predict the outcome, providing the user with a numeric estimate of the probability of that outcome for that patient or group of patients^12^.

The Acute Physiology and Chronic Health Evaluation (APACHE III), the Simplified Acute Physiology Score (SAPS II), and the Mortality Probability Model (MPM II) are among the most widely used prognostic models for outcome prediction in adult intensive care settings^13,14^.

ICUs have the highest death rate when compared to the hospital’s other ward services, even though mortality rates there vary based on the underlying disease^15^. The global average rate of mortality in ICUs ranges between 9 and 61%^16^. Multiple studies found that the ICU mortality rate varied around the world^16^.

Understanding the magnitude of intensive care unit (ICU) mortality and its associated predictors is essential for designing targeted interventions in the two selected government hospitals in Addis Ababa. Therefore, the present study aimed to assess the magnitude of mortality and identify its predictors among patients admitted to the ICUs at Tikur Anbessa Specialized Hospital (TASH) and Zewditu Memorial Hospital (ZMH) between December 1, 2023, and May 30, 2024.

## Method

### Study design and area

This study used an institution-based cross-sectional design and was conducted among ICU-admitted patients at Tikur Anbesa Specialized Hospital and Zewuditu Memorial Hospital.

### Source and study population

#### Inclusion Criteria

Patients were eligible for the study if they met all of the following conditions:

1. Clinically diagnosed and critically ill patients admitted to the ICU during the study period.
2. Age above 14 years.
3. Length of ICU stay was greater than 48 h.
4. Had complete medical records with documented allowances for the calculation of the Charlson Comorbidity Index (CCI).

## Exclusion Criteria

Patients were excluded from the study:

1. Duration of ICU stay less than 48 h.
2. Age ≤ 14 years.
3. Missing medical records.
4. Readmissions during the same study period.

### Sample size and procedure

To determine the minimum sample size for this study, we used the single-population proportion formula. For the sample size of the descriptive cross-sectional study, we used an expected prevalence of 29.6% from a study conducted in Ethiopian referral hospitals in 2023^17^. (Zα/2) the standard normal deviate at the 95% level of precision, the expected prevalence set at 29.6%, the acceptable margin of error set at 4%, and considering a 10% non-response, a missing medical chart. Based on this parameter, the total sample size was determined to be 309. Based on the allocation formula, the sample size was 209 for Tikur Anbesa Specialized Hospital and 100 for Zewditu Memorial Hospital.

### Data collection and quality assurance

Secondary data was collected from the charts of patients admitted to the ICU with a structured questionnaire. Data were collected using a structured questionnaire embedded in the Kobo Toolbox. The data collector was a trained physician to reduce errors and increase the overall reliability of the collected data. Periodic supervision by the principal investigator was conducted to oversee the process and maintain data quality.

### Measurement of variables

Charlson Comorbidity Index (CCI).

The Charlson Comorbidity Index was used to quantify patients’ comorbidity burden and predict mortality risk^18^.

The CCI scores for specific chronic conditions:

#### Score 1 (1 point each)

Myocardial infarction, congestive heart failure, peripheral vascular disease, cerebrovascular disease, dementia, chronic pulmonary disease, connective tissue disease, peptic ulcer disease, mild liver disease, diabetes mellitus (without end-organ damage).

#### Score 2 (2 points each)

Hemiplegia, moderate or severe renal disease, Diabetes with end-organ damage, any malignancy, Leukemia, Lymphoma.

#### Score 3 (3 points)

Moderate or severe liver disease.

#### Score 6 (6 points each)

Metastatic solid tumor and Acquired Immunodeficiency Syndrome (AIDS).

For age-adjusted CCI:

50–59 years: +1 point.

60–69 years: +2 points.

70–79 years: +3 points.

≥ 80 years: +4 points.

### Calculation method

The CCI score for each patient was calculated by adding:

The total score of each comorbid condition and the age adjustment score from the patient’s medical chart.

For final analysis, CCI was treated as a categorized variable (0, 1–2, 3–4, and ≥ 5).

### Statistical analysis

Before the analysis, the data were coded, cleaned, and checked for missing values. SPSS version 27 software was used to enter the data. Descriptive statistics were used. To evaluate the relationship between the exploratory factors and ICU mortality, bivariate and multivariable logistic regression analyses were used. For variables to be included in the multivariable logistic regression analysis, the p-value from the bivariate analysis must be less than 0.25. In multivariable analysis, variables were deemed statistically significant predictors of intensive care unit mortality if their p-values were less than 0.05 (AOR with 95% CI).

### Operational definition

#### Source of admission

patients admitted to the ICU from emergency, medical, surgical, and gynecology wards, or all.

#### Length of ICU stays

the number of days a patient stays in the ICU.

#### admission category

patients admitted to the ICU as surgical, medical, and gynecologic/obstetric.

#### Cause of death

an intermediate cause that led to the final event.

## Result

### A descriptive study of the participant

Over the course of six months, 626 patients were admitted. Of them, the charts of 309 sample patients were obtained. 100 patients’ charts were from ZMH, and the remaining 209 were from TASH. The respondents’ median age was 39. As shown in Table 1, most patients were between 21 and 40 years old. Of the patients, and 50.2% were male. Of the patients admitted, 44.3% were from the emergency department.

**Table 1:** Baseline socio-demographic profile of adult ICU patients admitted to two government hospitals in Addis Ababa, Ethiopia, 2024 (*n* = 309).

Variable	Category	Frequency	Percent
Age in the group in years	< 20	22	7.1%
	21–40	147	47.5%
	41 − 40	90	29.1%
	61–80	48	15.5%
	> 80	2	0.6%
Sex	Male	155	50.2%
	Female	154	49.8%
Location	TASH	209	67.4%
	ZMH	100	32.4%

Abbreviations: TASH; Tikur Anbesa Specialized Hospital, ZMH; Zewuditu Memorial Hospital.

As Table 2 shows, admission-related character: About 44.3% of patients were admitted from the emergency department, followed by the operating theater (23.6%) and medical ward (19.7%). Of this, the commonest causes of admission were postoperative, and the commonest causes of death were septic shock, stroke, head trauma, and ARDS. 141 (45.6%) of patients were mechanically ventilated.

**Table 2:** Distribution of ICU admission characteristics and primary diagnoses among patients admitted to two tertiary hospitals in Addis Ababa, Ethiopia, 2024 (*n* = 309).

Variable	Category	Frequency	Percent
Source of admission	Emergency department	137	44.3%
	Medical ward	61	19.7%
	Surgical ward	17	5.5%
	Operating theater	73	23.6%
	Gynecology/obstetrics	21	6.8%
Admission category	Medical patient	198	64.07
	Surgical patient	90	29.13%
	Gynecology/obstetrics	21	6.8%
Admission diagnosis	Myocardial infarction	8	2.6%
	Congestive heart failure	19	6.1%
	Septic shock	65	21%
	Pneumonia	9	2.9%
	ARDS	16	5.2%
	Pulmonary thromboembolism	3	1%
	Diabetic ketoacidosis	6	1.9%
	Stroke	24	7.8%
	Head trauma	16	5.2%
	General Surgical	80	25.9%
	CNS infection	22	7.1%
	GBS	3	1%
	Relapsing fever	7	2.3%
	Poisoning	4	1.3%
	Thyroid storm	3	1%
	Others	24	7.8%

Abbreviations: ARDS; Acute respiratory distress syndrome, CNS; Central Nervous System, GBS; Guillain-Barré Syndrome.

**Fig. 1:**
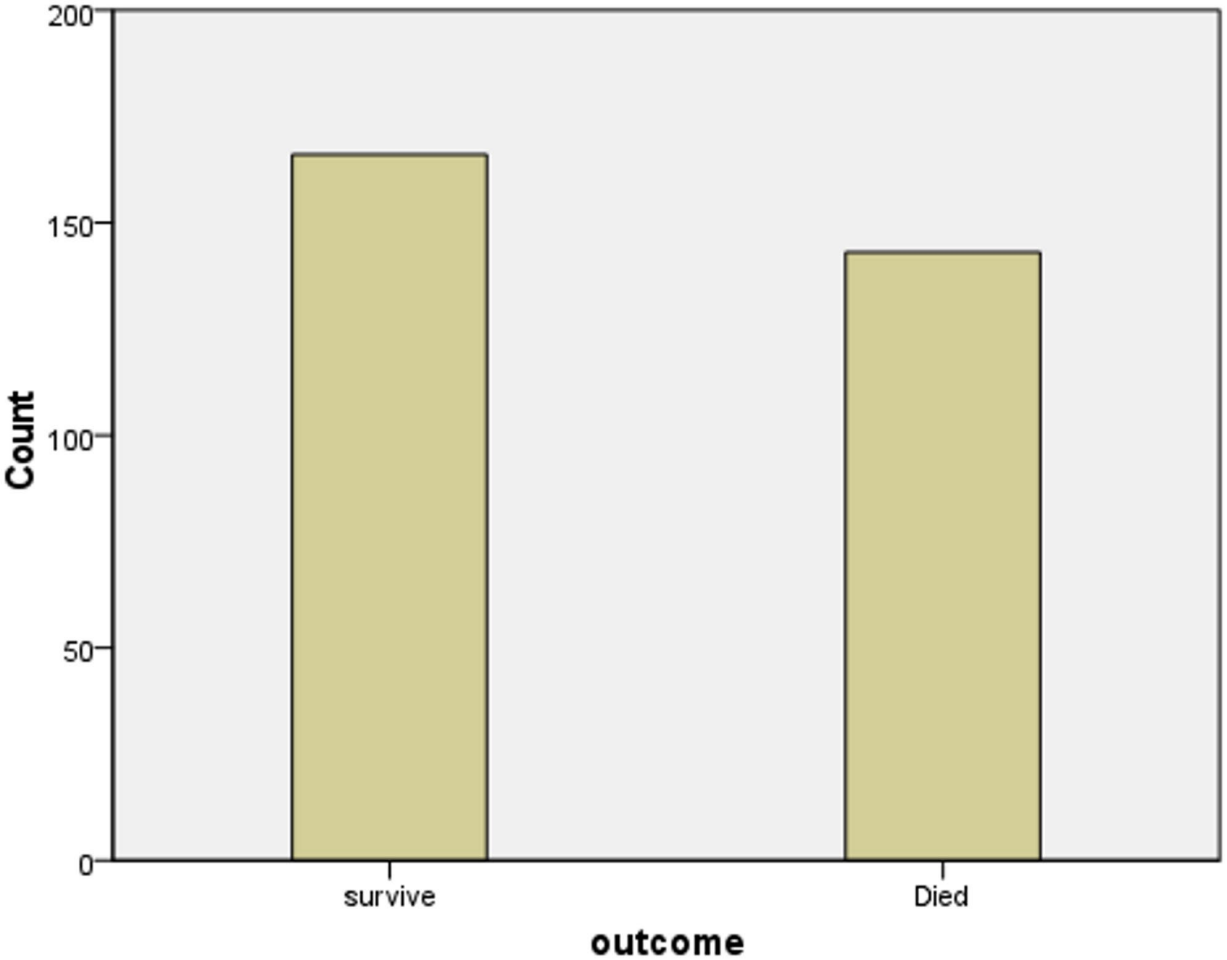
Number of ICU survivals and mortalities among study participants, 2024 (*n* = 309).

Figure 1 shows that 143 patients died during the study period. The overall prevalence of ICU mortality was 46.3%. The magnitude of death among patients admitted to ZMH was 46%, and TASH was 46.4%, which was comparable.

### Factors associated with ICU mortality

To find independent predictors of death among patients admitted to the critical care unit, logistic regression analysis was performed. To determine relationships between dependent and independent factors, five predictor variables with p-values < 0.25 in bivariate regression analyses were included in multivariable logistic regression analyses (Table 3) and (Table 4), respectively.

**Table 3:** Bivariate logistic regression analysis of factors associated with ICU mortality, 2024 (n = 309).

**Variable**	**Category**	**n (%)**	**AOR**	**95% CI**	**p-value**
Glasgow Coma Scale	9–12	28	6.7	1-3.6	<0.001
Need for Mechanical Ventilation	Yes	141	17.0	2.3–3.5	<0.001
Charlson Comorbidity Index Score	3–4	45	11.3	1.7–3.5	<0.001
	≥5	36	6.43	1.06–2.8	0.003
Hospital-Acquired Infection	Yes	60	115.9	3.5–22	<0.001
Acute Respiratory Distress Syndrome	Yes	16	8.89	1.03–21.55	0.004

In Table 4, which shows the multivariable logistic regression model, three variables, namely hospital-acquired infection, Charlson comorbidity index score, and mechanical ventilation required at admission, were significantly associated with ICU mortality at a p-value of 0.001.

**Table 4:** Multivariable logistic regression analysis of predictors of ICU mortality, 2024 (n = 309).

**Variable**	**Category**	**n **	**AOR (95% CI)**	**p-value**
Need for mechanical ventilation.	Yes	141	4.30 (3.10–6.28)	<0.001
Charlson Comorbidity Index score	≥3	198	2.20 (1.58–3.0 8)	<0.001
Hospital-acquired infection	Yes	60	2.90 (2.44–3.48)	<0.001

For those patients who had hospital-acquired infection, the odds of ICU mortality were 2.9 (AOR = 2.9; 95% CI: 2.442, 3.480) times higher than those who had no HAI (Table 4). Similarly, for patients who required mechanical ventilation at admission, the odds of ICU mortality were about 4.3 (AOR = 4.3; 95% CI: 3.1, 6.28) times higher than those who did not require mechanical ventilation at admission.

For patients with a Charlson comorbidity index ≥ 3 in the ICU, the odds of ICU mortality were approximately 2.2 times higher (AOR = 2.2; 95% CI: 1.58, 3.080) than those with a CCIS of less than 3 (Table 4).

## Discussion

This study determined the extent of mortality and its determinants among patients admitted to two government hospitals in Addis Abeba. The study found that the overall mortality rate was 46.3%. of hospitalized patients died in the ICU. This finding was consistent with the indigenous study by Hosanna, reporting 46.42%^19,20^. This finding was higher than that reported in studies in Nigeria (32.9%)^21^ and Singapore (26.5%)^22^. However, it was lower than previous studies in Ethiopia (67.4%)^23^, Kenya (53.6%)^24^, and Rwanda (47%)^20^. Several factors could account for these differences. Inadequate resources and infrastructure in many African countries may make timely and appropriate care difficult to obtain, leading to increased mortality.

Furthermore, the lack of a distinct ICU for surgical and medical patients in the research area, as well as the fact that the ZMH ICU is still a young facility, may contribute to the higher risk of ICU death. Differences in sample size, level of ICU care, availability of medical supplies, and classification of trained personnel could also explain the variance.

Our analysis identified three main predictors of ICU mortality: hospital-acquired infection, need for mechanical ventilation at admission, and a high Charlson Comorbidity Index (CCIS ≥ 3). All mentioned above independently increased the risk of death.

For patients who required mechanical ventilation at admission, the odds of ICU mortality were about 4.302 (AOR = 4.30 (3.10–6.28)) times higher than those who did not require mechanical ventilation at admission. which is consistent with study findings in Asian countries^25^. and Brazil^26^. Several factors could account for these differences. Inadequate resources and infrastructure in many African countries may make timely and appropriate care difficult to obtain, leading to increased mortality.

For patients with a Charlson comorbidity index > 3 in the ICU, the odds of ICU mortality were about 2.2 times higher (AOR = 1.56; 95% CI: 1.577, 3.080) than those with a CCIS < 2. Higher CCI is associated with increased ICU mortality, consistent with previous studies^27,28^. One such explanation is that patients with high CCI scores typically have multiple chronic conditions, which complicates their clinical picture and increases their susceptibility to acute infections. Comorbidities, such as Diabetes, heart disease, and chronic respiratory conditions, can influence how the body responds to stressors like trauma or infection, making it more difficult for people to recover from serious illnesses^29^.

For patients with hospital-acquired infection, the odds of ICU mortality were 2.9 (AOR = 2.9; 95% CI: 2.442, 3.480) times those of patients without HAI, consistent with the study’s finding in England^30^. HAIs frequently affect critically ill patients, worsening their overall health. The fact that infections can induce serious side effects like sepsis, which can overwhelm the body’s defenses and lead to multiple organ failure, may help to explain these findings. Additionally, by extending hospital stays and complicating treatment strategies, HAIs raise the risk of unfavorable outcomes.

The overall finding of this study underscores the importance of the Ethiopian government and organizations in providing intensive care services. The high prevalence of mortality rates shows that there is a need for an immediate policy response. Despite Ethiopia’s plan IV, the transformation focuses on critical care^31^. Translating policy into action is important, including staffing the ICU, ensuring standard equipment, and establishing benchmarks.

Second, the study identified three modifiable risk factors: high CCIS, HAI, and need for mechanical ventilation. This predictor indicates the need to develop a risk-stratification tool for the ICU. Third, the high HAI for mortality risk underscores the need to implement infection prevention and control guidelines with adequate resources and robust accountability mechanisms.

Fourth, high mortality among mechanically ventilated patients shows the need for basic intensive care training in mechanical ventilation. Increasing postgraduate programs in critical care medicine and respiratory therapy, and simulation-based training with international collaboration, will address basic staff knowledge of ICU care. The African Federation of Emergency Medicine highlights a workforce gap in sub-Saharan Africa, and Ethiopia must adopt this framework for better outcomes^32^.

Finally, the national clinical audit program should implement an ICU outcome monitoring mechanism, including the incidence of HAI and mechanical ventilator complications, using a tracking tool. Regular performance monitoring and feedback will improve ICU outcomes^33^.

## Conclusion

This study shows that ICU mortality in Ethiopia is high. Key warning signs include hospital-acquired infections, patients requiring mechanical ventilation, and a high Charlson comorbidity index. Overall, the study indicates the need for both clinical management and national critical care policies. By targeting these risks through focused quality improvement initiatives, it is possible to reduce ICU deaths and enhance patient recovery outcomes.

## Supplementary Information

Below is the link to the electronic supplementary material.

Supplementary Material 1

Supplementary Material 2

## Data Availability

The deidentified version of the dataset generated and analyzed during this study is provided within the supplementary documents.

## Abbreviations

AKI: Acute kidney injury
ARDS: acute respiratory distress syndrome
CCIS: Charlson comorbidity index score
ICU: Intensive Care Units
IHD: ischemic heart diseases
IRB: Institutional Review Board
LOS: length of stay
MV: mechanical ventilation
TASH: Tikur Anbesa Specialized Hospital
VAP: ventilator-associated pneumonia
ZMH: Zewuditu Memorial Hospital

## References

[1] Żerdziński, K., Janiec, J., Jóźwik, K., Łajczak, P. & Krzych, Ł. J. Artificial intelligence in intensive care: an overview of systematic reviews with clinical maturity and readiness mapping. *J. Clin. Med.* **15** (1), 185. 10.3390/jcm15010185 (2025). 41517434 10.3390/jcm15010185 PMC12786610

[2] Merola, R., Marra, A., Simone, S. D. & Vargas, M. Telemedicine in Intensive Care Unit: Current Practice and Future Prospect. *J. Intensive Care Med.* **40** (4), 456–463 (2025). doi:10.1177/08850666251325782 PubMed PMID: 40123239. 40123239 10.1177/08850666251325782

[3] Angelucci, A., Greco, M., Cecconi, M. & Aliverti, A. Wearable devices for patient monitoring in the intensive care unit. *Intensive Care Med. Exp.* **13** (1), 26. 10.1186/s40635-025-00738-8 (2025). 40016479 10.1186/s40635-025-00738-8 PMC11868008

[4] Ohbe, H. et al. Hospital and regional variations in intensive care unit admission for patients with invasive mechanical ventilation. *J. Intensive Care 2024 June* **5**;12:21. 10.1186/s40560-024-00736-0 PubMed PMID: 38840225; PubMed Central PMCID: PMC11155017. 10.1186/s40560-024-00736-0 PMC11155017 38840225

[5] Schell, C. O. et al. Hospital burden of critical illness across global settings: a point prevalence and cohort study in Malawi, Sri Lanka and Sweden. *BMJ Glob Health*. **10** (3), e017119. 10.1136/bmjgh-2024-017119 (2025). PubMed PMID: 40132811; PubMed Central PMCID: PMC12004492. 40132811 10.1136/bmjgh-2024-017119 PMC12004492

[6] Kilinc, M. Antibiotic Resistance and Mortality in ICU Patients: A Retrospective Analysis of First Culture Growth Results. *Antibiotics* **14** (3), 290. 10.3390/antibiotics14030290 (2025). 40149101 10.3390/antibiotics14030290 PMC11939337

[7] Bayraktar, Y. Ş. et al. Factors affecting mortality and clinical outcomes in intensive care unit patients with thoracic trauma: a retrospective, single-center study. *Medicina* **62** (2), 294. 10.3390/medicina62020294 (2026). 41752693 10.3390/medicina62020294 PMC12942034

[8] Métais, M. et al. Factors associated with ICU mortality and long-term outcomes in immunocompromised patients admitted to the intensive care unit for acute respiratory failure. *Ann. Intensive Care*. **15** (1), 175. 10.1186/s13613-025-01578-1 (2025). PubMed PMID: 41168567; PubMed Central PMCID: PMC12575889. 41168567 10.1186/s13613-025-01578-1 PMC12575889

[9] Cirik, M. O. et al. Comparison of intensive care scoring systems in predicting overall mortality of sepsis. *Diagnostics* **15** (13), 1660. 10.3390/diagnostics15131660 (2025). 40647659 10.3390/diagnostics15131660 PMC12248853

[10] Mușat, F. et al. Machine Learning Models in Sepsis Outcome Prediction for ICU Patients: Integrating Routine Laboratory Tests-A Systematic Review. *Biomedicines* **12** (12), 2892. 10.3390/biomedicines12122892 (2024). PubMed PMID: 39767798; PubMed Central PMCID: PMC11727033. 39767798 10.3390/biomedicines12122892 PMC11727033

[11] Sun, H., Kang, M., Zhang, H., Jia, J. & Wang, Q. Machine Learning for Predicting Mortality in Intensive Care Unit Patients: A Prognostic Performance Systematic Review and Meta-Analysis. *Nurs. Crit. Care*. **30** (6), e70206. 10.1111/nicc.70206 (2025). PubMed PMID: 41074688. 41074688 10.1111/nicc.70206

[12] Pellathy, T. P., Pinsky, M. R., Hravnak, M. & ICU Scoring Systems. *Crit. Care Nurse* ;**41**(4):54–64. (2021). doi:10.4037/ccn2021613 PubMed PMID: 34333619; PubMed Central PMCID: PMC8378550. 34333619 10.4037/ccn2021613 PMC8378550

[13] Jeong, S. Scoring Systems for the Patients of Intensive Care Unit. *Acute Crit. Care*. **33** (2), 102–104. 10.4266/acc.2018.00185 (2018). 31723870 10.4266/acc.2018.00185 PMC6849060

[14] Sekulic, A. D., Trpkovic, S. V., Pavlovic, A. P., Marinkovic, O. M. & Ilic, A. N. Scoring Systems in Assessing Survival of Critically Ill ICU Patients. *Med. Sci. Monit. Int. Med. J. Exp. Clin. Res. 2015 September* **4**;21:2621–2629. 10.12659/MSM.894153 PubMed PMID: 26336861; PubMed Central PMCID: PMC4562616. 10.12659/MSM.894153 PMC4562616 26336861

[15] Kalın, B. S., Özçaylak, S., Solmaz, İ. & Kılıç, J. Assessment of Risk Factors for Mortality in Patients in Medical Intensive Care Unit of a Tertiary Hospital. *Indian J. Crit. Care Med. Peer-Rev Off Publ Indian Soc. Crit. Care Med.* **26** (1), 49–52. 10.5005/jp-journals-10071-24092 (2022). PubMed PMID: 35110844; PubMed Central PMCID: PMC8783230. 10.5005/jp-journals-10071-24092 PMC8783230 35110844

[16] Endeshaw, A. S., Tarekegn, F., Bayu, H. T., Ayalew, S. B. & Gete, B. C. The magnitude of mortality and its determinants in Ethiopian adult intensive care units: A systematic review and meta-analysis. *Ann. Med. Surg.* **1**, 84:104810. 10.1016/j.amsu.2022.104810 (2022 December). 10.1016/j.amsu.2022.104810 PMC9793120 36582907

[17] Demass, T. B. et al. The magnitude of mortality and its predictors among adult patients admitted to the Intensive care unit in Amhara Regional State, Northwest Ethiopia. *Sci. Rep. 2023 July* **25**;13(1):12010. 10.1038/s41598-023-39190-7 PubMed PMID: 37491467; PubMed Central PMCID: PMC10368686. 10.1038/s41598-023-39190-7 PMC10368686 37491467

[18] Bai, X., Sun, L., Zhang, Y., Zhao, D. & Li, X. Charlson index predicts thirty-day mortality in critically ill stroke patients: MIMIC-IV retrospective study. *Sci. Rep.* **15** (1), 42341. 10.1038/s41598-025-26410-5 (2025). 41309807 10.1038/s41598-025-26410-5 PMC12661011

[19] Obsa, M. S., Adem, A. O. & Gete, G. B. Clinical outcomes of patients admitted in intensive care units of Nigist Eleni Mohammed Memorial Hospital of Hosanna, Southern Ethiopia. Int J Med Med Sci. 2017 June 30;9(6):79–85. 10.5897/IJMMS2017.1297

[20] Uwamariya, P. Reasons for admission in intensive care unit (ICU) and factors associated with poor outcome in referral public hospitals in Rwanda [Internet]. 2022 [cited 2024 January 12]. Available from: http://dr.ur.ac.rw/handle/123456789/1926

[21] Ilori, I. U. & Kalu, Q. N. Intensive care admissions and outcome at the University of Calabar Teaching Hospital, Nigeria. *J. Crit. Care* **27** (1), 105.e1–105.e4. 10.1016/j.jcrc.2011.11.011 (2012). PubMed PMID: 22304993. 10.1016/j.jcrc.2011.11.011 22304993

[22] Mukhopadhyay, A. et al. Risk Factors for Hospital and Long-Term Mortality of Critically Ill Elderly Patients Admitted to an Intensive Care Unit. *BioMed. Res. Int. 2014 Dec.* **162014**:e960575. 10.1155/2014/960575 10.1155/2014/960575 PMC4280808 25580439

[23] Seid, S., Adane, H. & Mekete, G. Patterns of presentation, prevalence and associated factors of mortality in ICU among adult patients during the pandemic of COVID 19: A retrospective cross-sectional study. *Ann. Med. Surg. 2012*. **77**, 103618. 10.1016/j.amsu.2022.103618 (2022). PubMed PMID: 35441008; PubMed Central PMCID: PMC9010017. 10.1016/j.amsu.2022.103618 PMC9010017 35441008

[24] Lalani, H. S. et al. Intensive Care Outcomes and Mortality Prediction at a National Referral Hospital in Western Kenya. *Ann. Am. Thorac. Soc.* **15** (11), 1336–1343. 10.1513/AnnalsATS.201801 (2018). -051OC PubMed PMID: 30079751. 30079751 10.1513/AnnalsATS.201801-051OC

[25] Rosenthal, V. D. et al. Risk factors for mortality over 18 years in 317 ICUs in 9 Asian countries: The impact of healthcare-associated infections. *Infect. Control Hosp. Epidemiol.* **44** (8), 1261–1266. 10.1017/ice.2022.245 (2023). 36278508 10.1017/ice.2022.245

[26] Junior, C. T., Franca, S. A., Okamoto, V. N., Salge, J. M. & Carvalho, C. R. R. Infection as an independent risk factor for mortality in the surgical intensive care unit. *Clinics* **68** (8), 1103–1108. 10.6061/clinics/2013(08)07 (2013). 24037005 10.6061/clinics/2013(08)07 PMC3752640

[27] Ofori-Asenso, R. et al. Effect of Comorbidity Assessed by the Charlson Comorbidity Index on the Length of Stay, Costs and Mortality among Older Adults Hospitalised for Acute Stroke. *Int. J. Environ. Res. Public. Health*. **15** (11), 2532. 10.3390/ijerph15112532 (2018). PubMed PMID: 30424531; PubMed Central PMCID: PMC6267000. 30424531 10.3390/ijerph15112532 PMC6267000

[28] Kim, D. H. et al. Age-adjusted Charlson comorbidity index score is the best predictor for severe clinical outcome in the hospitalized patients with COVID-19 infection. *Med. (Baltim).* **100** (18), e25900. 10.1097/MD.0000000000025900 (2021). PubMed PMID: 33951004; PubMed Central PMCID: PMC8104192. 10.1097/MD.0000000000025900 PMC8104192 33951004

[29] Zhang, Y. et al. Risk Factors for 28-Day mortality in a Surgical ICU: A Retrospective Analysis of 347 Cases. *Risk Manag Healthc. Policy*. **14**, 1555–1562 (2021). 303514 PubMed PMID: 33889038; PubMed Central PMCID: PMC8054819. 33889038 10.2147/RMHP.S303514 PMC8054819

[30] Vincent, J. L. et al. Prevalence and Outcomes of Infection Among Patients in Intensive Care Units in 2017. *JAMA* **323** (15), 1478–1487. 10.1001/jama.2020.2717 (2020). PubMed PMID: 32207816; PubMed Central PMCID: PMC7093816. 32207816 10.1001/jama.2020.2717 PMC7093816

[31] Federal Democratic Republic of Ethiopia. *Ministry of Health. Health Sector Transformation Plan IV (HSTP-IV)* (Ministry of Health, 2020).

[32] Bae, C. et al. Evaluating emergency care capacity in Africa: an iterative, multicountry refinement of the Emergency Care Assessment Tool. *BMJ Glob Health*. **3** (5), e001138. 10.1136/bmjgh-2018-001138 (2018). PMID: 30364370; PMCID: PMC6195145. 30364370 10.1136/bmjgh-2018-001138 PMC6195145

[33] Rowan, K. M. & Harrison, D. A. Recognising and responding to acute illness in patients in hospital. *BMJ* **335** (7631), 1165–1166. 10.1136/bmj.39395.497928.80 (2007). PMID: 18048503; PMCID: PMC2128622. 18048503 10.1136/bmj.39395.497928.80 PMC2128622

